# DOA Estimation Using Fourth-Order Cumulants in Nested Arrays with Structured Imperfections

**DOI:** 10.3390/s20040994

**Published:** 2020-02-12

**Authors:** Baoping Wang, Junhao Zheng

**Affiliations:** 1 National Key Laboratory of Science and Technology on UAV, Northwestern Polytechnical University, Xi’an 710072, China; 2 School of Electronics and Information, Northwestern Polytechnical University, Xi’an 710072, China; zjh1071218679@163.com

**Keywords:** nested arrays, mutual coupling, gain-phase errors, colored noise, fourth-order cumulants

## Abstract

Recently developed super nested array families have drawn much attention owing to their merits on keeping the benefits of the standard nested arrays while further mitigating coupling in dense subarray portions. In this communication, a new mutual coupling model for nested arrays is constructed. Analyzing the structure of the newly formed mutual coupling matrix, a transformation of the distorted steering vector to separate angular information from the mutual coupling coefficients is revealed. By this property, direction of arrival (DOA) estimates can be determined via a grid search for the minimum of a determinant function of DOA, which is induced by the rank reduction property. We also extend the robust DOA estimation method to accommodate the unknown mutual coupling and gain-phase mismatches in the nested array. Compared with the schemes of super nested array families on reducing the mutual coupling effects, the solutions presented in this paper has two advantages: (a) It is applicable to the standard nested arrays without rearranging the configuration to increase the inter-element spacing, alleviating the cross talk in dense uniform linear arrays (ULAs) as well as gain-phase errors in sparse ULA parts; (b) Perturbations in nested arrays are estimated in colored noise, which is significant but rarely discussed before. Simulations results corroborate the superiority of the proposed methods using fourth-order cumulants.

## 1. Introduction

Sensor array signal processing is a significant research area owing to its wide applications to radar, sonar, navigation, wireless communications, et al. [1,2,3,4]. Virtues of array signal processing, such as direction of arrival (DOA) estimation and beamforming, lie in spatial diversity and capability to determine the angular information of electromagnetic waves and enhance the reception of signals of interest while mitigating the interferences [5,6,7,8,9]. The basic but widely employed array geometry is the uniform linear array (ULA), where the inter-element spacing is usually less than or equal to half a wavelength to avoid spatial aliasing. However, the performance of ULAs is subject to a limited degrees of freedom (DOFs) that is linearly dependent on to the number of sensors. For instance, an *M*-elements ULA can offer up to M−1 DOFs, i.e., only M−1 signals can be resolved at most [10,11]. To increase the DOFs, a simple way is to introduce extra sensors, which may result in to difficulties in practical implementations. Besides, ULAs with a large number of sensors may suffer from prominent mutual coupling effects between physical sensors.

Sparse linear arrays (SLAs), a promising countermeasure, can tackle the above issues to a large extent. The critical operation on the DOFs enhancement is to vectorize the covariance matrix of the observations, rendering $O(M^{2})$ virtual sensors with *M* physical elements in the difference coarrays, which means one can probably resolve more DOAs than the physical sensors provided that there is no correlation between signals [12]. Compared with the ULA, this is a big leap in raising DOFs. In addition, the electromagnetic cross talk between sensors is alleviates since the inter-element spacing becomes larger in SLAs [13].

Recently, a new type of sparse array, so-called the nested array [14], is put forward in signal processing society and causes tremendous repercussions because the positions of sensors can be arranged in a regular design, the synthetic apertures becomes larger, and the resultant enhanced DOFs have a closed form expression with respect to the given number of sensors [15,16,17,18,19,20,21,22]. The nested array is constructed by cascading ULAs that differ in systematically designed inter-element spacing. In particular, the difference coarray formed from a two-level nested array has consecutive virtual sensors without any holes, while the hole-free property in the difference coarray cannot be guaranteed by higher level nested arrays that possess higher DOFs. The DOFs of nested arrays can be further increased by making the outer ULA much sparser, i.e., enlarging its aperture, as well as putting up an auxiliary sensor [21]. In [23,24], the one-dimensional nested array is extended to the two-dimensional case.

However, the mutual coupling effects of the nested array cannot be ignored at least as physical sensors in the inner ULA are deployed relatively close [25,26,27,28,29,30,31,32,33,34,35,36,37,38]. In [39], the super nested array in the context of the second-order statistics is designed to significantly mitigate the cross talk between sensors while preserving all advantages of the standard nested arrays, by increase the inter-element spacing of the inner ULA to maintain the coarray but alleviate the adverse electromagnetic effects. In [40], a high order super nested array is introduced to further reduce the mutual coupling between sensors. In [41], by splitting the inner ULA into two or four sections and rearranging them at the two sides of the remaining outer ULA, the so-called augmented nested array (ANA) is developed. Compared with the super nested array, the ANA can further reduce the unknown mutual coupling but at a cost of complicated design to guarantee the difference coarray hole-free. In [42], a new sparse array configuration, namely MISC array, is optimized to obtain maximum inter-element spacing, where a reasonable distribution of three sparse ULAs and two additional sensors is necessary. The MISC array is less susceptive to mutual coupling effects while achieving higher DOFs.

It should be noted that: (a) the aforementioned nested array configurations cannot fundamentally avoid the mutual coupling effects, i.e., all the DOA estimation methods performing on such arrays still work in the presence of perturbations, and (b) the existing nested array processing using the second-order statistics is vulnerable to colored noise. To obtain more robust estimates, we would deal with these two issues from another perspective—consider the array imperfections and jointly resolve DOAs and the parameterized perturbations—rather than reduce the errors by a new geometry design. This idea is reasonable in the sense that the super nested arrays and its successors occupying large areas are not applicable to some circumstances, such as airborne platforms, with a limited space for devices. In this correspondence, a new cumulant-based DOA estimation method for nested arrays with perturbations is proposed. The mutual coupling of the nested array is first analysed and parameterized in terms of the relationship of coupling range and inter-element spacing, giving a new structure of the mutual coupling matrix (MCM). Then we provide a proof that the distorted steering vector can be factorized into a new matrix, including angular information only, multiplied by the coupling coefficient vector. Leveraging this property, we develop a MUSIC-like estimator to determine the DOA estimates based on the rank reduction (RARE) technique in the context fourth-order cumulants (FOC). In addition, the proposed method can be extended to adapt to a more challenging scenario where mutual coupling and gain-phase errors coexist. Analytical specifications show that our solutions are more robust to array imperfections as compared to the standard nested array processing.

The remainder of this paper is organized as follows. In Section 2, an nested array model with unknown mutual coupling is introduced. In Section 3, the MUSIC-like estimators for nested arrays with perturbations using fourth-order cumulants are developed. Section 4 provides numerical examples for demonstrating the validity and efficiency of our proposed algorithms. Finally, some concluding remarks are given in Section 5.

## 2. Problem Formulation

### 2.1. Signal Model

Consider *N* narrowband non-Gaussian signals impinging on a linear *M*-elements nested array, composed of two different ULAs concatenating together. Assume that the inter-element spacing of the inner ULA with $M_{1}$ elements is $d_{I}$ and the inter-element spacing of the outer ULA with $M_{2}$ elements is $d_{O}$ satisfying $d_{O}=\left(M_{1}+1\right)d_{I}$. Without loss of generality, we set $d_{I}=\frac{\lambda }{2}$ with λ being the wavelength. To be specific, the sensors of a 2-level nested array are deployed at the positions that can be formulated as a union of the position set of inner ULA, $S_{I}$, and the position set of outer ULA, $S_{O}$, which can be expressed as

(1) $$\begin{array}{cc} P & =S_{I}\cup S_{O}\\ \; & =\left\{md_{I}|m=0,\cdots ,M_{1}-1\right\}\cup \left\{\left(n\left(M_{1}+1\right)+M_{1}\right)d_{I}|n=0,\cdots ,M_{2}-1\right\} \end{array}$$

If there exits mutual coupling between the array elements, the corresponding M×1 array observation vector is then given by

(2) $$\begin{array}{cc} x(t) & =\sum_{i=1}^{N}Ca\left(\theta_{i}\right)s_{i}\left(t\right)+n\left(t\right)=CAs\left(t\right)+n\left(t\right) \end{array}$$

where $a\left(\theta_{i}\right)={\left[\begin{array}{c} 1,e^{j\frac{2\pi p_{2}}{\lambda }\sin\theta_{i}},\cdots ,e^{j\frac{2\pi p_{M}}{\lambda }\sin\theta_{i}} \end{array}\right]}^{T}\in C^{M}$ is the steering vector, $A=\left[\begin{array}{c} a\left(\theta_{1}\right),\cdots ,a\left(\theta_{N}\right) \end{array}\right]$ is the array manifold, C denotes the MCM, and $s\left(t\right)={\left[\begin{array}{c} s_{1}\left(t\right),\cdots ,s_{N}\left(t\right) \end{array}\right]}^{T}$. We assume that the incident signals ${\left\{s_{i}\left(t\right)\right\}}_{i=1}^{N}$ are independent of each other, the noise vector n(t) is amenable to the Gaussian distribution, and it is independent of the signals. Besides, we assume that A is unambiguous, i.e., the steering vectors ${\left\{a\left(\theta_{i}\right)\right\}}_{i=1}^{N}$ are linearly independent for any set of distinct ${\left\{\theta_{i}\right\}}_{i=1}^{N}$.

As described in [27,28,29,30,31,32,33,34,35,36,37,38,43], it may reasonably consider that there are remarkable negative correlation between the coupling strength and the inter-element spacing, and the cross talk effects can be neglected if the distance between two sensors is larger than several times the length of the minimum inter-element spacing $d_{I}$. By assuming that the minimum inter-element spacing of two sensors without mutual coupling to be $Qd_{I}$, the generalized MCM can be approximated by

(3) $$C_{ij}=\left\{\begin{array}{cc} c_{|p_{i}-p_{j}|},\; & |p_{i}-p_{j}|\leq Qd_{I}\\ 0,\; & |p_{i}-p_{j}|>Qd_{I} \end{array}\right..$$

For the case of 2-level nested arrays, if $Q<M_{1}+1$, the MCM can be specifically expressed as

(4) $$\begin{array}{cc} C & =\left[\begin{array}{cccccccc} 1 & c_{1} & \cdots & c_{P-1} & \; & \; & \; & 0\\ c_{1} & 1 & c_{1} & \cdots & \ddots & \; & \; & \;\\ c_{2} & c_{1} & 1 & c_{1} & \cdots & c_{P-1} & \; & \;\\ \; & \ddots & c_{1} & 1 & \cdots & c_{P-2} & \; & \;\\ \; & \; & \ddots & \ddots & \ddots & \vdots & \; & \;\\ \; & \; & \; & \ddots & c_{1} & 1 & \; & \;\\ \; & \; & \; & \; & \; & \; & \ddots & \;\\ 0 & \; & \; & \; & \; & \; & \; & 1 \end{array}\right]\\ \; & =\mathrm{blkdiag}\left\{\mathrm{Toeplitz}\left\{\left[1,c_{1},\cdots ,c_{P-1},0_{1\times (M_{1}+1-P)}\right]\right\},I_{\left(M_{2}-1\right)}\right\} \end{array}$$

where $\mathrm{Toeplitz}\left\{r\right\}$ denotes a symmetric Toeplitz matrix constructed by the vector r, and $0<|c_{1}\left|,\right|c_{2}\left|,\cdots ,\right|c_{P-1}|<c_{0}=1$ are the mutual coupling coefficients.

### 2.2. Parameters Setting

To make the signal model (simulations) much easier to follow and replicate, it should be emphasized herein that throughout this communication, the following parameters are variables in the performance analysis and assessment: signal-to-noise ratio (SNR), the number of snapshots *L*, and the number of Monte Carlo runs; the fixed parameters adopted in the numerical calculus, deductions, and simulations include: the number of total sensors *M*, inter-element spacing of the inner ULA $d_{I}$, the number of sensors the inner ULA $M_{1}$, the number of sensors the outer ULA $M_{2}$, the number of signals *N*, DOAs ${\left\{\theta_{i}\right\}}_{i=1}^{N}$, the coupling range *P*, and the mutual coupling coefficients ${\left\{c_{i}\right\}}_{i=1}^{P-1}$.

## 3. Proposed FOC-Based DOA Estimator without Mutual Coupling Compensation

### 3.1. FOC Matrix Construction

Considering the received signals are assumed to be non-Gaussian, one can establish the array FOC matrix between the received data blocks as

(5) $$\begin{array}{cc} B & =\mathrm{cum}\left\{x\left(t\right),x^{H}\left(t\right),x^{*}\left(t\right),x^{T}\left(t\right)\right\} \end{array}$$

The entry of B in the $\left[\left(k_{1}-1\right)M+k_{2}\right]$-th row and the $\left[\left(l_{1}-1\right)M+l_{2}\right]$-th column is defined as

(6) $$\begin{array}{cc} \; & \;\;B\left(\left(k_{1}-1\right)M+k_{2},\left(l_{1}-1\right)M+l_{2}\right)\\ \; & =\mathrm{cum}\{x_{k_{1}}\left(t\right),x_{l_{1}}^{*}\left(t\right),x_{k_{2}}^{*}\left(t\right),x_{l_{2}}\left(t\right)\}\\ \; & =E\left[x_{k_{1}}\left(t\right)x_{k_{2}}^{*}\left(t\right)x_{l_{1}}^{*}\left(t\right)x_{l_{2}}\left(t\right)\right]-E\left[x_{k_{1}}\left(t\right)x_{k_{2}}^{*}\left(t\right)\right]E\left[x_{l_{1}}^{*}\left(t\right)x_{l_{2}}\left(t\right)\right]\\ \; & \;-E\left[x_{k_{1}}\left(t\right)x_{l_{1}}^{*}\left(t\right)\right]E\left[x_{k_{2}}^{*}\left(t\right)x_{l_{2}}\left(t\right)\right]-E\left[x_{k_{1}}\left(t\right)x_{l_{2}}\left(t\right)\right]E\left[x_{k_{2}}^{*}\left(t\right)x_{l_{1}}^{*}\left(t\right)\right] \end{array}$$

Substituting (2) into (5), and exploiting a series of properties of FOC in [44], one can further get

(7) $$\begin{array}{cc} B & =\mathrm{cum}\left\{CAs\left(t\right),{\left(CAs(t)\right)}^{H},{\left(CAs(t)\right)}^{*},{\left(CAs(t)\right)}^{T}\right\}\\ \; & =\mathrm{cum}\left\{\sum_{p=1}^{N}Ca\left(\theta_{p}\right)s_{p}\left(t\right),\sum_{m=1}^{N}{\left(Ca(\theta_{m})\right)}^{H}s_{m}^{*}\left(t\right),\sum_{q=1}^{N}Ca^{*}\left(\theta_{q}\right)s_{q}^{*}\left(t\right),\sum_{n=1}^{N}{\left(Ca(\theta_{n})\right)}^{T}s_{n}\left(t\right)\right\}\\ \; & =\sum_{p=1}^{N}\sum_{m=1}^{N}\sum_{q=1}^{N}\sum_{n=1}^{N}\left(Ca\left(\theta_{p}\right)⊗{\left(Ca(\theta_{q})\right)}^{*}\right){\left(Ca\left(\theta_{m}\right)⊗{\left(Ca(\theta_{m})\right)}^{*}\right)}^{H}\mathrm{cum}\left\{s_{p}\left(t\right),s_{m}^{*}\left(t\right),s_{q}^{*}\left(t\right),s_{n}\left(t\right)\right\}\\ \; & =\sum_{i=1}^{N}\left(Ca\left(\theta_{i}\right)⊗{\left(Ca(\theta_{i})\right)}^{*}\right){\left(Ca\left(\theta_{i}\right)⊗{\left(Ca(\theta_{i})\right)}^{*}\right)}^{H}\mathrm{cum}\left\{s_{i}\left(t\right),s_{i}^{*}\left(t\right),s_{i}^{*}\left(t\right),s_{i}\left(t\right)\right\}\\ \; & =\sum_{i=1}^{N}\gamma_{i}\left(Ca\left(\theta_{i}\right)⊗{\left(Ca(\theta_{i})\right)}^{*}\right){\left(Ca\left(\theta_{i}\right)⊗{\left(Ca(\theta_{i})\right)}^{*}\right)}^{H}\\ \; & =\left(CA\circ {\left(CA\right)}^{*}\right)C_{s}{\left(CA\circ {\left(CA\right)}^{*}\right)}^{H} \end{array}$$

where $\gamma_{i}≜\mathrm{cum}\left\{s_{i}\left(t\right),s_{i}^{*}\left(t\right),s_{i}^{*}\left(t\right),s_{i}\left(t\right)\right\}$, and $C_{s}≜\mathrm{diag}\left\{\gamma_{1},\cdots ,\gamma_{N}\right\}\in R^{N\times N}$.

To resolve the DOA estimates from the FOC built above, some approaches [45,46,47,48] have been proposed. However, these methods are developed for ULAs in the presence of unknown mutual coupling, and cannot be directed applied to the case of nested arrays as the mutual coupling effects of the two array geometries are distinct from each other. By this reason, a new estimator using FOC should be investigated for combating the unknown mutual coupling.

### 3.2. Robust DOA Estimation Against Unknown Mutual Coupling

By examining the special structure of the MCM of the nested arrays, one finds that the actual steering vector can be factorized in another way by

(8) $$\begin{array}{cc} Ca(\theta ) & =T(\theta )c \end{array}$$

where $c=\left[1,c_{1},\cdots ,c_{P-1}\right]\in C^{P}$, and $T\left(\theta \right)=\left[\begin{array}{c} T_{1}+T_{2}\\ T_{3} \end{array}\right]\in C^{M\times P}$ where $T_{1}$, $T_{2}\in C^{\left(M_{1}+1\right)\times P}$, and $T_{3}\in C^{\left(M_{2}-1\right)\times P}$ are given by

(9) $${\left[T_{1}\right]}_{p,q}=\left\{\begin{array}{cc} {\left[a_{1}\right]}_{p+q-1},\; & p+q\leq M_{1}+2\\ 0,\; & \mathrm{otherwise} \end{array}\right.$$

(10) $${\left[T_{2}\right]}_{p,q}=\left\{\begin{array}{cc} {\left[a_{1}\right]}_{p-q+1},\; & p\geq q\geq 2\\ 0,\; & \mathrm{otherwise} \end{array}\right.$$

(11) $$\begin{array}{cc} T_{3} & =\left[a_{2},0_{\left(M_{2}-1\right)\times \left(P-1\right)}\right] \end{array}$$

with $a_{1}=F_{1}a\left(\theta \right)$, $F_{1}=\left[I_{M_{1}+1},0_{\left(M_{1}+1\right)\times \left(M_{2}-1\right)}\right]$, $a_{2}=F_{2}a\left(\theta \right)$, and $F_{2}=\left[0_{\left(M_{2}-1\right)\times \left(M_{1}+1\right)},I_{M_{2}-1}\right]$.

**Proof** **of** **(8).** One can first rewrite the left-hand side of (8) as

(12) $$\begin{array}{cc} Ca(\theta ) & =\left[\begin{array}{cc} C_{1} & 0\\ 0 & I_{M_{2}-1} \end{array}\right]\left[\begin{array}{c} a_{1}\\ a_{2} \end{array}\right]=\left[\begin{array}{c} C_{1}a_{1}\\ a_{2} \end{array}\right] \end{array}$$

where $C_{1}=\mathrm{Toeplitz}\left\{\left[1,c_{1},\cdots ,c_{P-1},0_{1\times (M_{1}+1-P)}\right]\right\}\in C^{\left(M_{1}+1\right)\times \left(M_{1}+1\right)}$. By Lemma 3 in [25], $C_{1}a_{1}=\bar{T}\left(\theta \right)c$ where $\bar{T}\left(\theta \right)=T_{1}+T_{2}$. Considering the first entry of **c** is 1, one can make an identical transformation between $a_{2}$ and **c**, that is, $a_{2}=\left[a_{2},0_{\left(M_{2}-1\right)\times \left(P-1\right)}\right]c=T_{3}c$. As a result, one has $Ca\left(\theta \right)=\left[\begin{array}{c} \left(T_{1}+T_{2}\right)c\\ T_{3}c \end{array}\right]=\left[\begin{array}{c} T_{1}+T_{2}\\ T_{3} \end{array}\right]c=T\left(\theta \right)c$. This completes the proof of (8). □

Now performing the singular value decomposition (SVD) on B, one has

(13) $$\begin{array}{cc} B & =U\Sigma V^{H} \end{array}$$

where $\Sigma =\mathrm{diag}\left\{\lambda_{1},\cdots ,\lambda_{M^{2}}\right\}$ consists of $M^{2}$ singular values satisfying $\lambda_{1}\geq \cdots \geq \lambda_{N}>\lambda_{N+1}=\cdots =\lambda_{M^{2}}=0$. The columns of $U_{s}≜U\left(:,1:N\right)$ are the singular vectors corresponding to the *N* largest eigenvalues, while the columns of $U_{n}≜U\left(:,N+1:M^{2}\right)$ are the singular vectors corresponding to the $M^{2}$ singular values which are all zeros. The signal subspace is spanned by the columns of $U_{s}$, whereas the noise subspace is spanned by the columns of $U_{n}$. One can construct the following function

(14) $$\begin{array}{cc} p\left(\theta ,c\right) & ={∥{\left(\left(Ca(\theta )\right)⊗{\left(Ca(\theta )\right)}^{*}\right)}^{H}U_{n}∥}_{2}^{2}\\ \; & ={∥{\left(\left(T(\theta )c\right)⊗{\left(T(\theta )c\right)}^{*}\right)}^{H}U_{n}∥}_{2}^{2}\\ \; & ={\left(c⊗c^{*}\right)}^{H}{\left(T\left(\theta \right)⊗T^{*}\left(\theta \right)\right)}^{H}U_{n}U_{n}^{H}\left(T\left(\theta \right)⊗T^{*}\left(\theta \right)\right)\left(c⊗c^{*}\right)\\ \; & =g^{H}\left(c\right)M\left(\theta \right)g\left(c\right) \end{array}$$

where $g\left(c\right)=c⊗c^{*}\in C^{P^{2}}$, and $M\left(\theta \right)={\left(T\left(\theta \right)⊗T^{*}\left(\theta \right)\right)}^{H}U_{n}U_{n}^{H}\left(T\left(\theta \right)⊗T^{*}\left(\theta \right)\right)\in C^{P^{2}\times P^{2}}$. Next one can estimate DOAs from the determinant M(θ). It can be found that the size of ${\left(T\left(\theta \right)⊗T^{*}\left(\theta \right)\right)}^{H}U_{n}$ is $P^{2}\times \left(M^{2}-N\right)$ and if $P^{2}\leq M^{2}-N$, the matrix ${\left(T\left(\theta \right)⊗T^{*}\left(\theta \right)\right)}^{H}U_{n}$ is normally of full row rank and M(θ) is of full rank. However, when θ is equal to any DOA of interest, i.e., $\theta =\theta_{i},i=1,\cdots ,N$, Equation (14) is zero according to the orthogonality between the signal and noise subspaces. Since $g\left(c\right)\neq 0$, (14) is available only if the matrix M(θ) is rank deficient, i.e., the determinant of M(θ) is zero. Hence, one can determine DOA estimates by searching the minimum of $\det\left\{M(\theta )\right\}$ that is close to zero as

(15) $$\begin{array}{cc} \hat{\theta } & =\arg\min_{\theta }\det\left\{M(\theta )\right\}. \end{array}$$

### 3.3. Mutual Coupling Coefficient Estimation

With the DOA estimates, the orthogonality between the signal and noise subspaces can be exploited to estimate the mutual coupling coefficients. To this end, one can stack a series of equations as

(16) $$\begin{array}{cc} \left[\begin{array}{c} U_{n}^{H}\left(\left(Ca({\hat{\theta }}_{1})\right)⊗{\left(Ca({\hat{\theta }}_{1})\right)}^{*}\right)\\ \vdots \\ U_{n}^{H}\left(\left(Ca({\hat{\theta }}_{N})\right)⊗{\left(Ca({\hat{\theta }}_{N})\right)}^{*}\right) \end{array}\right]\; & =\left[\begin{array}{c} 0_{\left(M^{2}-N\right)\times 1}\\ \vdots \\ 0_{\left(M^{2}-N\right)\times 1} \end{array}\right]. \end{array}$$

Substituting (8) into (16), one gets

(17) $$\begin{array}{cc} \underset{H}{\underbrace{\left[\begin{array}{c} U_{n}^{H}\left(T\left({\hat{\theta }}_{1}\right)⊗T^{*}\left({\hat{\theta }}_{1}\right)\right)\\ \vdots \\ U_{n}^{H}\left(T\left({\hat{\theta }}_{N}\right)⊗T^{*}\left({\hat{\theta }}_{N}\right)\right) \end{array}\right]}}\left(c⊗c^{*}\right)\; & =\left[h,\bar{H}\right]\left[\begin{array}{c} 1\\ \bar{c} \end{array}\right]=0_{N\left(M^{2}-N\right)\times 1}. \end{array}$$

where h is the first column of H while $\bar{H}$ contains the remaining columns of H, and $\bar{c}=\left[0_{\left(P^{2}-1\right)\times 1},I_{P^{2}-1}\right]\left(c⊗c^{*}\right)\in C^{P^{2}-1}$.

One can carry out the estimation of $\bar{c}$ in the least squares sense, that is

(18) $$\begin{array}{cc} \bar{c} & =-{\bar{H}}^{+}h. \end{array}$$

Then the mutual coupling coefficients $c_{1}=\left[c_{1},c_{2}\cdots ,c_{P-1}\right]$ can be extracted from $\bar{c}$ by

(19) $$\begin{array}{cc} c_{1} & ={\bar{F}}_{1}{\bar{c}}^{*} \end{array}$$

(20) $$\begin{array}{cc} c_{1} & ={\bar{F}}_{2}\bar{c} \end{array}$$

where ${\bar{F}}_{1}=\left[I_{P-1},0_{\left(P-1\right)\times \left(P^{2}-P\right)}\right]$ and ${\bar{F}}_{2}={\left[e_{P},e_{2P},\cdots ,e_{P(P-1)}\right]}^{T}$ with $e_{i}\in R^{P^{2}-1}$, i=P,2P,⋯,P(P−1), is a column vector with 1 at the *i*-th entry and 0 elsewhere. Finally, an improved estimate of $c_{1}$ can be obtained by averaging the above results as

(21) $$\begin{array}{cc} c_{1} & =\frac{1}{2}\left({\bar{F}}_{1}{\bar{c}}^{*}+{\bar{F}}_{2}\bar{c}\right). \end{array}$$

### 3.4. Extension to Partly Calibrated Nested Array with Unknown Mutual Coupling

In this section, the nested array is partly calibrated, i.e., the inner ULA suffers from the mutual coupling effect while the outer ULA has unknown gain and phase errors.

In practice, one may encounter an even more challenging scenario where the mutual coupling and gain-phase mismatch coexist in the nested array. However, it is difficult or even not able to get the issue solved as there are too many unknowns to be handled by the limited DOFs. To make DOA estimation feasible, one has to relieve the nuisance imperfections on the array to some extend, by calibrating a portion of sensors well. This class of arrays, referred to as partly calibrated nested arrays, is common in practice as the aperture of the inner ULA is typically much smaller than that of the whole array and, hence, the inner ULA can be assumed to be well calibrated for gain-phase error while the calibration of the outer ULA may be poor due to unknown channel imbalances between sensors that are located far away from each other. Through above analysis, the MCM is rectified as

(22) $$\begin{array}{cc} \bar{C} & =\mathrm{blkdiag}\left\{\mathrm{Toeplitz}\left\{\left[1,c_{1},\cdots ,c_{P-1},0_{1\times (M_{1}+1-P)}\right]\right\},\Upsilon_{\left(M_{2}-1\right)\times \left(M_{2}-1\right)}\right\} \end{array}$$

where $\Upsilon =\mathrm{diag}\left\{\beta \right\}$ with $\beta ={\left[\beta_{1},\beta_{2},\cdots ,\beta_{M_{2}-1}\right]}^{T}\in C^{M_{2}-1}$ containing the element gains ${\left|\beta_{i}\right|}_{i=1}^{M_{2}-1}$ and phases ${\left\{\arg\left(\beta_{i}\right)\right\}}_{i=1}^{M_{2}-1}$. Following a similar idea, one can derive

(23) $$\begin{array}{cc} \bar{C}a\left(\theta \right) & =\bar{T}\left(\theta \right)\alpha \end{array}$$

where $\alpha ={\left[c^{T},\beta^{T}\right]}^{T}$ and $\bar{T}\left(\theta \right)=\mathrm{blkdiag}\left\{T_{1}+T_{2},{\bar{T}}_{3}\right\}\in C^{M\times \left(P+M_{2}-1\right)}$ with ${\bar{T}}_{3}=\mathrm{diag}\left\{\left[e^{j\frac{2\pi p_{M_{1}+2}}{\lambda }\sin\theta },\cdots ,e^{j\frac{2\pi p_{M}}{\lambda }\sin\theta }\right]\right\}$. To prove (23), one needs to prove $\Upsilon a_{2}={\bar{T}}_{3}\beta$ that can be easily verified by the fact that $\mathrm{diag}\left\{b\right\}d=\mathrm{diag}\left\{d\right\}b$ holds for arbitrary vectors b and d with the same size. Then, by the same principle behind (13)–(15), one can determine the DOA estimates by

(24) $$\begin{array}{cc} \hat{\theta } & =\arg\min_{\theta }\det\left\{\bar{M}\left(\theta \right)\right\}, \end{array}$$

where $\bar{M}={\left(\bar{T}\left(\theta \right)⊗{\bar{T}}^{*}\left(\theta \right)\right)}^{H}U_{n}U_{n}^{H}\left(\bar{T}\left(\theta \right)⊗{\bar{T}}^{*}\left(\theta \right)\right)\in C^{{\left(P+M_{2}-1\right)}^{2}\times {\left(P+M_{2}-1\right)}^{2}}$.

Following the same procedures (16)–(21) and denoting $\bar{\alpha }=\left[0_{\left({\left(P+M_{2}-1\right)}^{2}-1\right)\times 1},I_{{\left(P+M_{2}-1\right)}^{2}-1}\right]\left(\alpha ⊗\alpha^{*}\right)\in C^{{\left(P+M_{2}-1\right)}^{2}-1}$, one can get the estimate of $\bar{\alpha }$ by

(25) $$\begin{array}{cc} \bar{\alpha } & =-{\tilde{H}}^{+}\bar{h}. \end{array}$$

where $\bar{h}$ is the first column of $\overset{˘}{H}$ that is defined as

(26) $$\begin{array}{cc} \overset{˘}{H} & =\left[\begin{array}{c} U_{n}^{H}\left(\bar{T}\left({\hat{\theta }}_{1}\right)⊗{\bar{T}}^{*}\left({\hat{\theta }}_{1}\right)\right)\\ \vdots \\ U_{n}^{H}\left(\bar{T}\left({\hat{\theta }}_{N}\right)⊗{\bar{T}}^{*}\left({\hat{\theta }}_{N}\right)\right) \end{array}\right], \end{array}$$

and $\tilde{H}$ includes the rest columns of $\overset{˘}{H}$. The final mutual coupling coefficient and gain-phase error estimates are determined by $\bar{\alpha }$ as

(27) $$\begin{array}{cc} c_{1} & =\frac{1}{2}\left({\tilde{F}}_{1}{\bar{\alpha }}^{*}+{\tilde{F}}_{2}\bar{\alpha }\right) \end{array}$$

(28) $$\begin{array}{cc} \beta & =\frac{1}{2}\left({\tilde{F}}_{3}{\bar{\alpha }}^{*}+{\tilde{F}}_{4}\bar{\alpha }\right) \end{array}$$

where ${\tilde{F}}_{1}=\left[I_{P-1},0_{\left(P-1\right)\times \left({\left(P+M_{2}-1\right)}^{2}-P\right)}\right]$, ${\bar{F}}_{2}={\left[{\bar{e}}_{P+M_{2}-1},{\bar{e}}_{2\left(P+M_{2}-1\right)},\cdots ,{\bar{e}}_{\left(P-1\right)\left(P+M_{2}-1\right)}\right]}^{T}$, ${\tilde{F}}_{3}=\left[0_{\left(M_{2}-1\right)\times \left(P-1\right)},I_{M_{2}-1},0_{\left(M_{2}-1\right)\times \left({\left(P+M_{2}-1\right)}^{2}-P-M_{2}+1\right)}\right]$, and ${\bar{F}}_{4}=\left[{\bar{e}}_{P\left(P+M_{2}-1\right)},{\bar{e}}_{\left(P+1\right)\left(P+M_{2}-1\right)},\cdots ,\right.{\left.{\bar{e}}_{\left(P+M_{2}-2\right)\left(P+M_{2}-1\right)}\right]}^{T}$ with ${\bar{e}}_{i}\in R^{{\left(P+M_{2}-1\right)}^{2}-1}$, $i=P+M_{2}-1,2\left(P+M_{2}-1\right),\cdots ,\left(P+M_{2}-2\right)\left(P+M_{2}-1\right)$, is a column vector with 1 at the *i*-th entry and 0 elsewhere.

The major steps of the proposed algorithm are summarised in Table 1.

**Remark** **1.** *The methods presented in this paper are valid on condition that the MCM is isolated from any angular information of the incident signals and are not applicable to arbitrary geometries of antenna arrays. However, they still work for some commonly used antenna arrays like dipole, monopole, and slot arrays [49]. On the other hand, the MCM could be direction variant for directional antenna arrays. In such a case, the proposed methods preform well as long as the MCM possesses a similar structure to (4) or (22), even if the mutual coupling coefficients $c_{1}$ are direction-dependent. This is because the transformation of product of the nominal steering vector and the direction-independent MCM still holds for the direction-dependent MCM by (8) or (23). To be specific, the array observation vector, of size M×1, can be expressed as $x\left(t\right)=\left[C_{1}a\left(\theta_{1}\right),C_{2}a\left(\theta_{2},\cdots ,C_{N}a\left(\theta_{N}\right)\right)\right]s\left(t\right)+n\left(t\right)$ where $C_{i}$ is reliant upon $\theta_{i}$, different from each other in general. It should be noted that if ${\left\{C_{i}\right\}}_{i=1}^{N}$ have the same banded symmetric Toeplitz structure as shown in (4), then one still has $C_{i}a\left(\theta_{i}\right)=T\left(\theta_{i}\right)c_{i}$. As a result, one can readily deduce that $det\left\{M(\theta )\right\}=0$ holds for $\theta =\theta_{i},i=1,2,\cdots ,N$, and the DOA estimates can be obtained by searching the minimum of $det\left\{M(\theta )\right\}$. It should be noted that the structure of MCM of directional antenna arrays may not stay the same for all angles in practice, causing an arbitrariness on the composition of M(θ) and, hence our solution is inapplicable to arbitrary direction-dependent mutual coupling via searching the spatial spectrum of a unified function $det\left\{M(\theta )\right\}$. Only the direction-independent model is discussed herein while its direction-dependent counterpart will be investigated in the future.*

**Remark** **2.** *As with the previous work [49], the coupling range P in (4) is assumed to be known* a priori*, otherwise it has to be determined properly. If P is selected to be smaller than the true value, the remaining mutual coupling may still deteriorate the estimation performance, whereas if the chosen P is larger than the true one, the accuracy and resolution of estimates are confined due to the loss of array aperture. A pragmatic approach to identify P is to measure the minimal distance between two antennas where the coupling effect is negligible. In practice, the coupling range depends on various factors of antenna design, such as array geometry, ground structure, material characteristic, etc, and the effect of mutual coupling is weak enough to be neglected in many applications when the inter-element spacing of an antenna array is larger than half a wavelength, which means that P is relatively small.*

**Remark** **3.** *It should be noted that one may get some pseudo estimates in addition to the true DOAs, satisfying (15) or (24). Without loss of generality, we assume that there are $N_{0}\geq N$ DOA estimates, pseudo and true, one obtains by a spectral search of (15) or (24). Substituting the DOA estimates into (21) or (27), on can determine $N_{0}$ mutual coupling coefficients vectors and reconstruct the corresponding MCM ${\left\{{\hat{C}}_{i}\right\}}_{i=1}^{N_{0}}$. Defining a spatial spectrum equation as follows*

(29) $$\begin{array}{cc} f & ={∥{\left(\left({\hat{C}}_{i}a\left({\hat{\theta }}_{i}\right)\right)⊗{\left({\hat{C}}_{i}a\left({\hat{\theta }}_{i}\right)\right)}^{*}\right)}^{H}U_{n}∥}_{2}^{2},\;i=1,2,\cdots ,N_{0}, \end{array}$$

one can sift the estimates of true DOAs and mutual coupling coefficients from the pseudo ones by identifying whether f=0 or not as the orthogonality between the signal subspace $\left({\hat{C}}_{i}a\left({\hat{\theta }}_{i}\right)\right)⊗{\left({\hat{C}}_{i}a\left({\hat{\theta }}_{i}\right)\right)}^{*}$ and the noise subspace $U_{n}$ does not hold for the false DOAs and mutual coupling coefficients. In practice, only finite snapshots are available, so the true DOA and mutual coupling coefficient estimates correspond to the minima of (29) that returns large values for the rest $N_{0}-N$ pseudo estimates. By this means, one can tell the true estimates from the false ones.

**Remark** **4.** It is known that ${\left(T\left(\theta \right)⊗T^{*}\left(\theta \right)\right)}^{H}U_{n}\in C^{P^{2}\times \left(M^{2}-N\right)}$ and ${\left(\bar{T}\left(\theta \right)⊗{\bar{T}}^{*}\left(\theta \right)\right)}^{H}U_{n}\in C^{{\left(P+M_{2}-1\right)}^{2}\times \left(M^{2}-N\right)}$, so the proposed method works on condition that $P^{2}\leq M^{2}-N$ for the case of unknown mutual coupling only and ${\left(P+M_{2}-1\right)}^{2}\leq M^{2}-N$ for the scenario where unknown mutual coupling and gain-phase error occur on dense ULAs and sparse ULA parts, respectively. This provides upper bounds on the number of resolvable signals for these two circumstances: one is $N\leq M^{2}-P^{2}$, and the other is $N\leq M^{2}-{\left(P+M_{2}-1\right)}^{2}$, which means that the developed algorithm can handle more signals in the former than in the latter, or equivalently, more DOFs and effective aperture are available when mutual coupling takes place only. Therefore, the proposed Method 1 has better performance than Method 2 in that intuitively, the more the unknowns are, the worse the estimation arises, while a reverse phenomenon can be observed since Method 1 does not take the gain-phase error into consideration, causing a considerable model mismatch.

## 4. Simulation Results and Discussion

In this section, various numerical experiments under different conditions are carried out to examine the performance of the proposed algorithms. Simulations are conducted for 2-level nested array with four elements in each level. For simplicity, we assume that all signals have an identical power. Similar to the settings in [50,51], the signals are modeled as s(t)=F(t)r(t), where $F\left(t\right)=\mathrm{diag}\left\{f_{1}\left(t\right),\cdots ,f_{K}\left(t\right)\right\}$, $r\left(t\right)={\left[r_{1}\left(t\right),\cdots ,r_{K}\left(t\right)\right]}^{T}$ with $f_{i}\left(t\right)$ and $r_{i}\left(t\right)$ being zero-mean Gaussian processes with unit-variance and $\sigma_{s}^{2}$-variance, respectively. The noise is assumed to be a first order spatial autoregressive process, and the (a,b)-th entry of the noise covariance matrix is given by $R\left(a,b\right)=\sigma_{n}^{2}0.8^{|\frac{p_{a}-p_{b}}{\lambda }|}$ [52,53]. The SNR is defined as $\mathrm{SNR}=10\log_{10}\left(\sigma_{s}^{2}/\sigma_{n}^{2}\right)$. The accuracy of the DOA estimate, the statistical performance of the algorithms, is measured from 800 Monte Carlo runs in terms of the root mean square error (RMSE) which is defined as

(30) $$\begin{array}{cc} {\mathrm{RMSE}}_{\theta } & =\sqrt{\frac{1}{800N}\sum_{n=1}^{800}\sum_{i=1}^{N}{\left({\hat{\theta }}_{i}^{\left(n\right)}-\theta_{i}\right)}^{2}} \end{array}$$

(31) $$\begin{array}{cc} {\mathrm{RMSE}}_{c} & =\sqrt{\frac{1}{800{∥c_{1}∥}_{2}^{2}}\sum_{n=1}^{800}{∥{\hat{c}}_{1}^{\left(n\right)}-c_{1}∥}_{2}^{2}}\times 100\; \end{array}$$

where ${\hat{\theta }}_{i}^{\left(n\right)}$ and ${\hat{c}}_{1}^{\left(n\right)}$ are the estimates of $\theta_{i}$ and $c_{1}$, respectively, for the *n*-th trial, and *N* is the number of signals.

**Figure 1:**
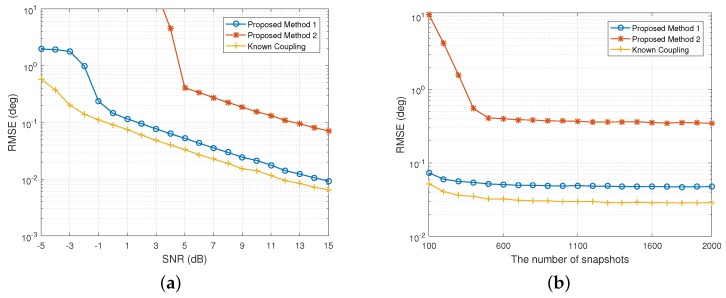
RMSE of the DOA estimates of 2 independent signals versus (**a**) SNR when N=500; (**b**) *N* when SNR=5 dB.

In the first scenario, we consider that two independent sources from $\left[-17^{\circ },43^{\circ }\right]$ impinge on the nested array. The resultant RMSEs of DOA estimates are illustrated in Figure 1. It can be seen that the proposed Method 1 approaches the FOC-based estimator with known coupling asymptotically and significantly outperforms the Method 2 with the increase of SNR. The main reason is that Method 1 deals with the outer ULA as calibrated and, hence more DOFs are obtained for DOA estimation, whereas Method 2 considers a even worse case, where the outer ULA has gain-phase errors, and the corresponding DOFs are occupied by the nuisance parameters. Besides, the estimation error of Method 2 decreases clearly as the number of snapshots increases and stabilize at approximately $0.35^{\circ }$ with the observation size larger than 500 snapshots, while both Method 1 and 4-MUSIC provide much better accuracy, saturating at RMSEs of $0.05^{\circ }$ and $0.03^{\circ }$, respectively, than Method 1 through all snapshot sizes when SNR is fixed at 5 dB.

Table 2 and Table 3 list the RMSEs of the mutual coupling coefficient estimates under different SNRs and snapshot sizes. Similar to the performance of DOA estimation in the first scenario, the above tabular results show that the proposed Method 1 is able to offer satisfactory estimation of mutual coupling, especially for moderate SNRs and the numbers of snapshots. A plausible explanation is that compared with Method 2, using the calibrated subarray has implicit superiorities, such as a lower Cramer-Rao lower bound for mutual coupling estimation, since its unknowns are less than those with the partly calibrated array. As a result, Method 1 can achieve better performance on mutual coupling calibration.

## 5. Conclusions

In this paper, an FOC-based DOA estimation method for nested arrays is proposed to simultaneously improve the robustness of direction finding of non-Gaussian signals to array imperfections and mitigate the spatially colored noise. Under the assumptions that the mutual coupling effects are direction-independent and its range is known *a priori*, the mutual coupling effects of nested arrays is analytically specified from the aspect of the relationship between coupling strength and inter-element spacing, and a new factorization of the distorted steering vector into a matrix, containing bearing information only multiplied by the coupling coefficient vector. Combining this property with RARE technique, a MUSIC-like estimator is developed to obtain the DOA estimates in FOC. Additionally, the proposed estimator can be extended to handle a much harsher issue that mutual coupling and gain-phase mismatches separately occur on dense and sparse sunbarrays, respectively. Analytical specifications show that our solutions are more robust to array imperfections as compared to the standard nested array processing. Compared with the schemes of super nested array families on reducing the mutual coupling effects, our solutions enjoy two merits: (a) It is applicable to the standard nested arrays without rearranging the configuration to increase the inter-element spacing, isolating the cross talk in dense subarrays as well as gain-phase errors in sparse ULA parts; (b) Perturbations in nested arrays are estimated in colored noise, which has not been fully addressed. Simulations results corroborate that the proposed methods are advantageous to self array calibration as well as immunity to colored noise.

## Figures and Tables

**Table 1:** Summary of the proposed method against the perturbations of nested arrays.

**Step 1.**	Compute the cumulant matrix **B** from observations x(t) according to (5).
**Step 2.**	Extract the noise subspace $U_{n}$ by performing SVD on **B**.
**Step 3.**	Construct $T\left(\theta \right)$ (or $\bar{T}\left(\theta \right)$) composed by $T_{1}$ in (9), $T_{2}$ in (22),
	and $T_{3}$ in (11) (or ${\bar{T}}_{3}$ in (23)).
**Step 4.**	Determine the DOA estimates ${\left\{{\hat{\theta }}_{i}\right\}}_{i=1}^{N}$ by searching for *N*
	minima of $det\left\{M(\theta )\right\}$ defined in (14) (or $\bar{M}\left(\theta \right)$ in (24)).
**Step 5.**	Reconstruct H with the DOA estimates ${\hat{\theta }}_{i}$ in (17) (or $\overset{˘}{H}$ in (26)).
**Step 6.**	Estimate $\bar{c}$ in (18) (or $\bar{\alpha }$ in (25)).
**Step 7.**	Obtain estimates of the mutual coupling coefficients ${\hat{c}}_{1}$ in (21)
	(or ${\hat{c}}_{1}$ in (27) and estimates of gain-phase errors $\hat{\beta }$ in (28)).

**Table 2:** Root mean square error (RMSE) of $c_{1}$ versus SNR when L=500.

	Method 1	Method 2
**SNR**	**RMSE** ${}_{c}$	**RMSE** ${}_{c}$
5 dB	8.534%	35.94%
7 dB	5.621%	23.413%
9 dB	3.7062%	13.881%
11 dB	2.5199%	8.2154%
13 dB	1.7612%	4.9845%
15 dB	1.2811%	3.2019%

**Table 3:** RMSE of $c_{1}$ versus the number of snapshots when SNR=5 dB.

	Method 1	Method 2
**The Number**	**RMSE** ${}_{c}$	**RMSE** ${}_{c}$
**of Snapshots**		
1000	8.1907%	35.118%
1200	8.0853%	34.504%
1400	8.0204%	34.884%
1600	8.0126%	34.465%
1800	7.9647%	34.305%
2000	7.9368%	34.043%
